# Supplementary material for: Field-free Magnetization Switching by Utilizing the Spin Hall Effect and Interlayer Exchange Coupling of Iridium
Source: Sci Rep. 2019 Jan 23;9:325. doi: 10.1038/s41598-018-37586-4 (PMC6344501; doi:10.1038/s41598-018-37586-4)
Supplement: Supplementary file 1 — Supplementary information [file 41598_2018_37586_MOESM1_ESM.pdf]

Supplementary Information

Field-free Magnetization Switching by Utilizing the Spin Hall Effect and Interlayer Exchange Coupling of Iridium

Yang Liu[1,3], Bing Zhou[1,3], and Jian-Gang (Jimmy) Zhu[2,3,*]

[1] Department of Materials Science and Engineering, Carnegie Mellon University, Pittsburgh, Pennsylvania, 15213, USA

[2] Department of Electrical and Computer Engineering, Carnegie Mellon University, Pittsburgh, Pennsylvania, 15213, USA

[3] Data Storage Systems Center, Carnegie Mellon University, Pittsburgh, Pennsylvania, 15213, USA

* jzhu@cmu.edu

1. Interlayer exchange coupling of Iridium (Ir)

Film stacks for investigating the interlayer exchange coupling of Ir are substrate/ Co (2 nm)/ Ir (t_{Ir})/ Co (2 nm)/ Ta (2 nm) where t_{Ir} ranges from 0.6 nm to 1.65 nm. In-plane hysteresis loops were measured by alternating gradient field magnetometer (AGFM) and used to determine the strength of interlayer exchange coupling. Figure S1 shows the exchange coupling field as a function of Ir layer thickness. Note that only antiferromagnetic coupling is observed in our experiments. As can be seen, the first antiferromagnetic coupling peak locates at 0.6 nm and the second at 1.35 nm. For the magnetization switching experiments, we choose the Ir layer thickness to be 1.35 nm for that the exchange coupling via 0.6 nm Ir is so strong that the perpendicular Co layer can be pulled into in-plane direction by its coupled in-plane Co layer.

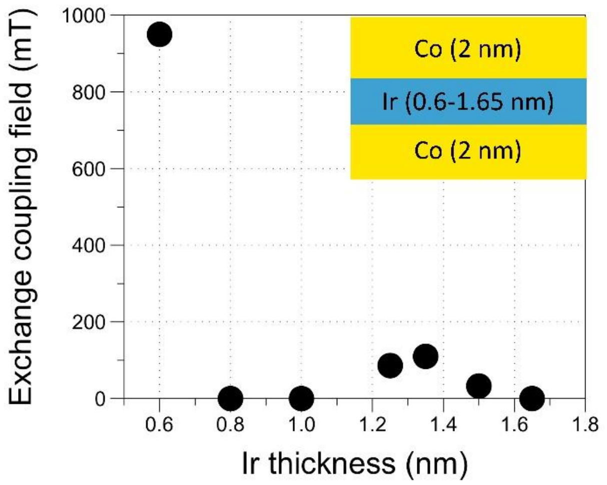

FIG. S1. Exchange coupling field as a function of Ir layer thickness

2. Spin Hall effect (SHE) induced magnetization switching in the device without the in-plane magnetized layers.

We also fabricated the devices that are of the film stack without the bottom in-plane magnetized layers, that is Substrate/ Ta (0.5 nm)/ Ir (3 nm)/ Co (1.2nm)/ Ta (2nm). SHE induced magnetization switching measurements were conducted and the results are shown in Figure S2. In this case, no observable switching is obtained in absence of an external field. On the other hand, magnetization reversal is observed with external field $H_x = \pm 10$ mT. The switching loops are flipped when reversing the in-plane field direction. This is a typical behavior of SHE induced switching for that the preferred magnetization state depends on the in-plane field direction.

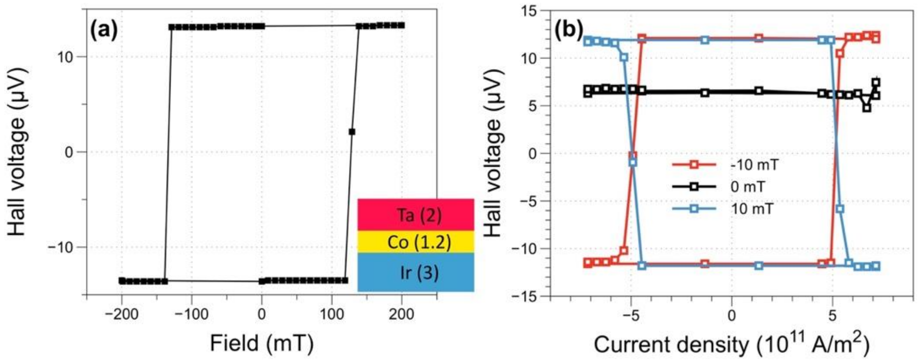

FIG. S2. (a) Anomalous Hall effect loop of the device without the bottom in-plane Co layers. (b) Current induced magnetization switching under different external fields.

3. Spin Hall effect (SHE) induced magnetization switching in the device with a thick Ir layer.

Now the stack contains a 3 nm-thick Ir layer, as shown in Figure S3. With this thickness, the strength of interlayer exchange coupling should decay to nearly zero. Results shows that in this case magnetization reversal can only occur with applying an external field H_x. This partially proves the field-free magnetization switching presented in the main body of manuscript is facilitated by the in-plane field resulting from the interlayer exchange coupling via the 1.35 nm Ir.

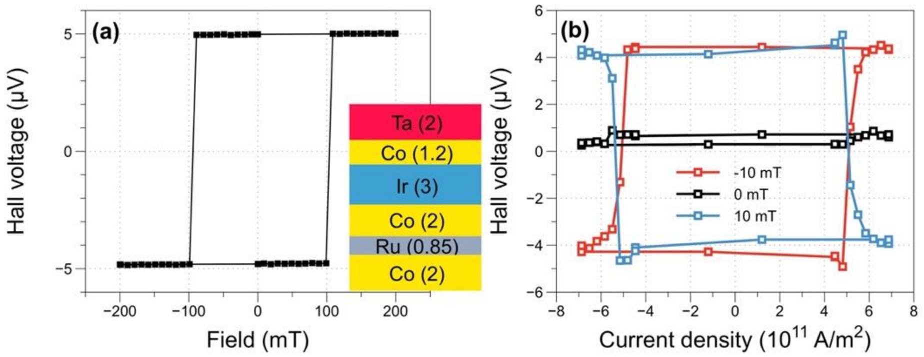

FIG. S3. (a) Anomalous Hall effect loop of the device with a 3 nm Ir layer. (b) Current induced magnetization switching under different external fields.

4. Domain wall chirality and effective DMI field at Ir/Co interface

To characterize the domain wall chirality as well as to measure the effective DMI field at Ir/Co interface, the growth of magnetic bubble domains was measured with Kerr microscope. During this measurement, a magnetic bubble domain was first initiated. Then perpendicular field ($H_z = 10$ mT) pulses were generated to grow the bubble domain, in the presence of an in-plane field H_x. The growing dynamics was recorded and used for analyzing the growth velocity of up-down and down-up domain walls. A summary of domain wall velocity as a function of in-plane field is plotted as Figure S4. Typically, higher domain wall velocity is favored when H_x is parallel to the direction of effective DMI field within the domain wall[1,2]. Thus, our observations show that the domain wall in our films is right-handed Neel-type wall. The two minima in domain wall velocity plot indicates the effective DMI field is balanced with H_x when they are antiparallel. It implies that the effective DMI field at Ir/Co interface is around 9 mT, much smaller than that at Pt/Co[1] and W/FeCoB interface[3]. As the antiparallel H_x further increases and overcomes the effective DMI field, it reverses the domain wall chirality such that the domain wall moments are aligned with H_x. So beyond the minima, domain wall velocity starts to increase with larger H_x.

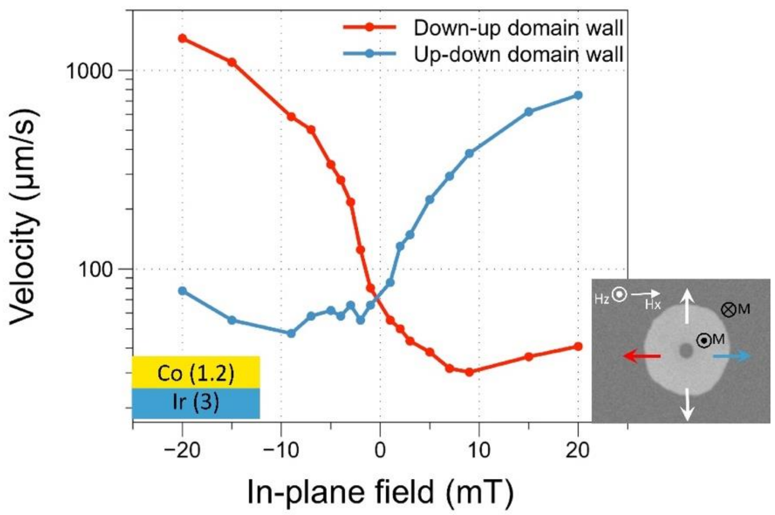

FIG. S4. Velocity of down-up and up-down domain wall as a function of in-plane magnetic field. This plot shows the chirality of domain wall at Ir/Co interface is right-handed. The effective DMI field is about 9 mT.

References

1. Lau, D., Sundar, V., Zhu, J. G. & Sokalski, V. Energetic molding of chiral magnetic bubbles. *Phys. Rev. B* **94,** 1–5 (2016).

2. Je, S. G. *et al.* Asymmetric magnetic domain-wall motion by the Dzyaloshinskii-Moriya interaction. *Phys. Rev. B - Condens. Matter Mater. Phys.* **88,** 1–5 (2013).

3. Liu, Y., Liu, X. & Zhu, J.-G. Tailoring the Current-Driven Domain Wall Motion by Varying the Relative Thickness of Two Heavy Metal Underlayers. *IEEE Trans. Magn.* (2018).
